# Leveraging structure-informed machine learning for fast steric zipper propensity prediction across whole proteomes

**DOI:** 10.1371/journal.pcbi.1013395

**Published:** 2025-08-25

**Authors:** Samantha Zink, Songrong Qu, Thomas Holton, Eesha Shankar, Paulina Stanley, David S. Eisenberg, Michael R. Sawaya, Jose A. Rodriguez

**Affiliations:** 1 Department of Chemistry and Biochemistry; UCLA-DOE Institute for Genomics and Proteomics, STROBE, NSF Science and Technology Center, University of California, Los Angeles (UCLA), Los Angeles, California, United States of America; 2 Departments of Chemistry and Biochemistry and Biological Chemistry, Howard Hughes Medical Institute, UCLA-DOE Institute, Molecular Biology Institute, UCLA, Los Angeles, California, United States of America; University of Kansas, UNITED STATES OF AMERICA

## Abstract

Predicting the amyloid fold and the propensity of peptide segments to adopt amyloid-like structures remain a challenge. However, recent progress has facilitated structure-based prediction of steric zipper propensity and the use of machine learning to accelerate the calculation of predictive models across many scientific areas. Leveraging these advances, we have developed a new approach for rapid proteome-wide assessment of zipper profiles that is informed by four million steric zipper predictions collected over ten years. This collection is used to build a machine learning model capable of rapidly predicting steric zipper propensity, and allowing for the assessment of zippers at both the protein and proteome level. Our predictions show enrichment for zipper forming segments in proteins involved in cell wall reorganization in yeast, highlighting a potential category of interest for experimental characterization. Overall, our predictive model allows for the exploration of amyloid formation across the tree of life and provides a tool for assessment of both novel and designed sequences for zipper density.

## Author summary

The amyloid fold of proteins is described by formations of un-branched, beta-sheet-rich fibrils, and is observed in a number of pathogenic and non-pathogenic processes in living organisms. Adhesive segments termed ‘steric zippers,’ which involve pairs of sheets mating at a tight and relatively dry interface, have been hypothesized to drive amyloid formation. The online database tool ZipperDB has freely enabled computing the propensity for short peptide segments to form zippers. Training on the results of queries submitted since its inception, we now create a structure-informed machine learning model that faithfully replicates the prediction process in accelerated fashion. This tool allows for zipper scores to be computed for all possible hexapeptide sequences, and proteome-wide analyses of zipper propensity to be determined and analyzed, leading to an improved understanding of their putative distributions across proteomes.

## Introduction

The amyloid fold appears across the tree of life. While many amyloids are associated with protopathic diseases [1], non-pathogenic amyloids have emerged as a counterpoint to the idea that amyloid assemblies result from misfolding errors. Non-pathogenic amyloids can be functional, or fulfill a specific role; for example, these can range from structural to regulatory [2]. Ongoing efforts have significantly broadened our understanding of the universe of amyloid proteins, but our perspective of amyloid-forming proteins or domains across entire proteomes is still limited.

Despite their unique functions or associations with disease, amyloidogenic proteins are defined by a set of structural features that are common to all amyloid folds: They form higher order assemblies, typically fibrils that are typically elongated, un-branched, and composed of individual protein monomers folded in roughly 2-dimensional layers, stacked ~ 4.8Å apart [3]. Amyloid fibrils can be composed of thousands of monomer layers. Stacked β-sheets run parallel and in-register to the fibril axis while extended protein strands outspread perpendicularly from the axis. This scaffold is the source of the diagnostic cross-β diffraction pattern observed in pathogenic amyloids.[ [4,5] In amyloid fibrils, stacked β-strands are stabilized through backbone hydrogen bonding which extend the length of the fibril. In amyloid fibril cores, the interfaces of mating β-sheets show tight interdigitation of peptide side chains, typically excluding water molecules from their core. This structural motif was termed the steric-zipper, and is thought to be a major contribution to the stability of amyloid fibrils [6].

Most proteins contain segments predicted to form steric zippers, and, in principle, any protein could adopt an amyloid fold. This ubiquity of putative zippers makes it challenging to predict amyloid propensity from sequence alone. That challenge is demonstrated by the diversity of amyloid-prone sequences can be seen in the set of ~30 known amyloid proteins [3]. However, in specific cases, the concentration of putative zippers can dictate the amyloid core of a protein, as is the case for prions [7]. Some categories of proteins have a unique sequence composition that might predispose proteins to form functional amyloids; this is the case with prions. An enrichment of branched polar residues (Q/N) is a hallmark of prion-like proteins [7]. This has allowed for the development of sequence-based machine learning (ML) approaches, such as that developed by Alberti et al., that resulted in the identification of 29 new yeast prion candidates that matched a particular sequence profile [8]. It is currently not known, however, if the unique link between sequence composition and prion or amyloid folds extends beyond yeast prions or if this is diagnostic of functional prion behavior across organisms. Beyond the specific case of prion proteins, identifying benign or pathogenic proteins with amyloid-forming capabilities remains crucial, both for understanding disease mechanisms and for characterizing novel protein sequences.

Structure-based approaches are an alternative method of identifying novel sequences that are predicted to adopt an amyloid-like fold. The online zipper database (zipperDB) is an example of structure-based amyloid predictors [9]. Instead of only looking at the sequence, zipperDB models six residue segments of a protein sequence onto a type 1 steric zipper backbone and uses the Rosetta energy to evaluate the likelihood of that segment's ability to adopt a steric zipper [9]. This approach has successfully identified segments prone to forming zippers in the lab, and has been used to query millions of hexapeptide sequences. However, its dependence on a multi-parameter search and optimization necessarily makes zipperDB computationally expensive and thus limits the scale of its deployment and the investigation of zipper forming potential on a proteome level.

Here, we aim to leverage the pre-existing scores generated by zipperDB over its 10 + years of existence by using machine learning to dramatically accelerate the assessment of potential amyloid-forming sequences. We train a fully-connected neural network on 4.1 million zipperDB sequence-score pairs and pre-compute zipper propensity scores for all possible 64 million hexapeptide sequences. We then investigate the utility of using type-1 zipper prediction scores to identify protein domains or regions with putative amyloid-forming potential across entire proteomes. This tool is publicly available as a web-server (https://zipperdb.mbi.ucla.edu) that uniquely enables the exploration of zipper forming potential across the tree of life.

## Results

### ML-based prediction of hexapeptide zipper propensities

**Fig 1:**
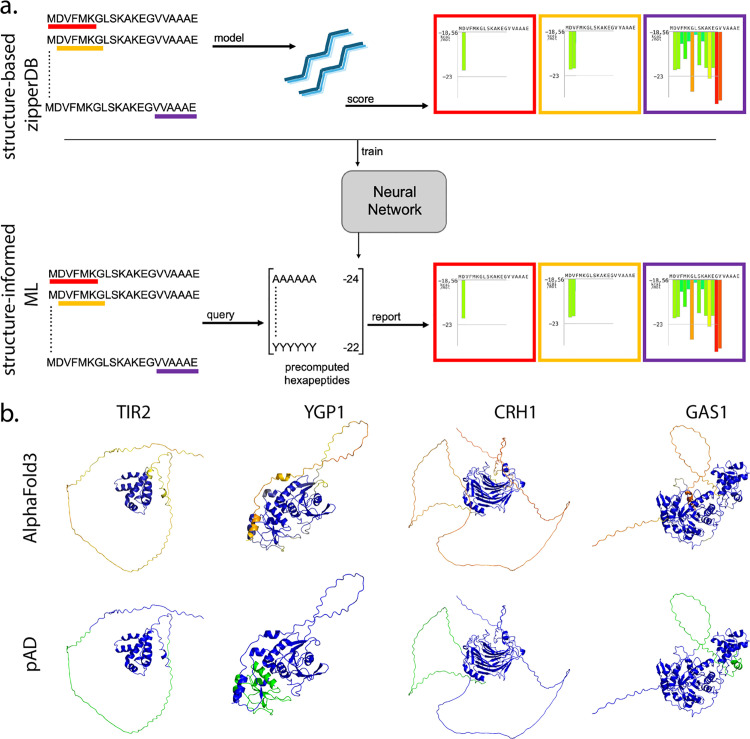
Structure-informed machine learning-based steric zipper prediction. **a.)** Comparison of structure-based 3D profiling method to structure-informed machine learning-based approach. Top panel shows structure-based zipperDB methodology. Sequence is divided into hexapeptide segments, individually modeled onto NNQQNY backbone where the energetics are evaluated and finally are reported in the zipper profile. A decade of data from this process (4.1 million hexapeptide sequence/score pairs) were collected and fed into a neural network which then produced scores for all 64 million possible hexapeptide sequences. The structure-informed method will query the precomputed table to produce the zipper profile. **b.)** Candidate examples from S. cerevisiae proteome. AlphaFold3 models are shown on top colored by pLDDT scores (blue > 90, yellow < 50). Bottom panel shows pAD highlighted in green.

**Fig 2:**
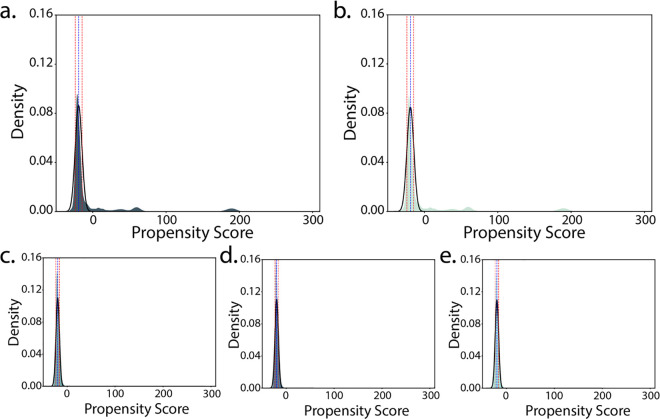
Score distribution histograms for zipper data subsets. Histograms were plotted across all five datasets: **a.**) all hexapeptides (allHex), **b.**) sequences excluded from the machine learning set (excl) and machine learning sets, **c.**) training, **d.**) validation, and **e.**) test. The means for scores < 0 across all five sets exhibited overlapping ranges (allHex = -19.27 ± 4.62, excluded = -19.23 ± 4.7, train = -19.79 ± 3.62, validation = -19.79 ± 3.62, test = -19.81 ± 3.6).

We trained a neural network on a subset of 4.1 million hexapeptide sequence-score pairs that were originally generated via the structure-based threading method of zipperDB (Fig 1a) [9]. We then used this network to generate scores for all possible 64 million hexapeptides and performed a thorough assessment on the features of that data set. We first explored the properties of the full set of 64 million possible hexapeptides, compared to the sets used in the training of the neural network. We separated the data into groups: all possible hexapeptides (allHex, n = 64M), the machine-learning set, which includes all segments submitted to the original zipperDB (train = 3.28M, test = 0.134M, and validation = 0.733M), and the set of hexapeptides excluded from training (excluded = 59.86M). The hexapeptide scores are related to ΔG, where a negative score, reported in Rosetta Energy Units (REU), indicates increased stability and a more favorable arrangement. Histograms of the scores are multimodal in the allHex and excluded sets, with a majority of the density falling between -50 and 0 REU (Fig 2a-2b). Two additional sub-populations are apparent: one centered around 180 REU and one present between 0 and 75 REU. These populations represent hexapeptides that, when modeled in Rosetta, exhibit either unrealistically large scores indicative of physically impossible clashes or reveal potential biases in the scoring function for specific residues. The histograms from each machine learning set (train/test/validation) appear unimodal with most of the data falling between -50 and 0 REU (Fig 1c-1e). For scores < 0, the averages across all five datasets are overlapping, indicating agreement across sets included and excluded from training. The differences in modality are likely due to bias in in the original zipperDB toward protein sequences suspected to form amyloid; the sequences that constitute the training data were selected by users interested in amyloid, likely reducing sequence diversity. Therefore, the training data likely does not exhibit the diversity in all possible hexapeptide sequences, but we expected the volume of data to sufficiently mitigate the present biases.

We then assessed the behavior of the hexapeptide scores across various sequence compositions. In the cases of hydrophobic and polar residues, an increase in the number of the specific amino acid type leads to a more favorable zipper score. As the number of hydrophobic residues in the hexapeptide increases, there is a higher likelihood of adopting a steric zipper motif, as reported by a linear decrease in the propensity score (S2a Fig). We see the same linear dependency in the number of β-branched residues (I, T, V) present in the hexapeptide (S2f Fig); as the number of β-branched residues in a putative zipper segment increase, its propensity score becomes more favorable. Finally, the number of polar residues present is correlated to an increase in zipper propensity (S2c Fig). Polar residues contain additional hydrogen bonding capabilities through their side chain atoms, which are thought to further stabilize the steric zipper motif. These trends are true across all five data subsets.

Other amino acid types are correlated to a decrease in zipper propensity, as is the case with an increase in the number of aromatic and charged residues. Aromatic residues are thought to stabilize the steric zipper through π-π stacking van der Waals (VDW) interactions. In our analysis, however, we see a miniscule shift to less favorable as the number of aromatic residues in the hexapeptide is increased (< 1 REU) (S2b Fig). Additionally, there is a decrease in zipper propensity when adding charged residues (both negative and positive) as well as special residues (C, G, P) to the hexapeptide sequence (S3 and S4 Figs). Charged residues tend to destabilize the zipper arrangement due to electrostatic repulsion and/or unmatched charge complementarity. Sequences containing proline residues were omitted from the original zipperDB due to their unusually high propensity scores. Our method similarly yields elevated scores for proline-containing sequences, leading us to regard these scores as unreliable. These sequences drive the decrease in zipper propensity seen with the addition of special residues (S3 Fig).

To understand this further, we examined how charge influences the propensity score of a hexapeptide, independent of its amino acid composition. As the total number of charged residues increases, the average propensity score becomes less favorable (S4 Fig). This is also true when looking at the total charge of the hexapeptide. The resulting data takes on a parabolic shape with the minima at zero total charge and increasing as the total charge moves towards ± 6 (S4 Fig). Interestingly, there is a linear increase in score when adding charged residues to both the zipper interface and the solvent interface (S5 Fig). We would expect adding charged residues to the solvent interface to have less of a detrimental effect to the score than adding a charge to the closely packed, dry zipper interface. This highlights potential biases in the solvation parameters used for initial zipper scoring.

Lastly, we assessed how zipper propensity changes when each amino acid occupies each position in the hexapeptide. We took the average score of all hexapeptides that had the amino acid of interest in the position of interest and expanded across all amino acids and positions in the hexapeptide. A majority of the amino acids had average scores of ~ -20 REU across all positions in the hexapeptide with the exceptions of arginine and proline (S6 Fig). Scores of arginine-containing hexapeptides remained stable constant across positions, regardless of the arginine position; however, its average score of ~ -15 REU was higher than other residue types. Arginine is a large, positively charged amino acid and is not expected to pack well in the zipper interface. Additionally, proline scores were inconsistent across positions. This is likely due to limitations in the training data as the original zipperDB did not routinely include scores for prolines. Additionally, the sequences with prolines that were included in training were inconsistent and scored very high, indicating Rosetta had difficulty in modeling proline residues in the type-1 zipper backbone. In conclusion, arginine was a consistent driver in unfavorable scores and the inconsistency of proline scores made it unreliable.

### Assessment of predicted zipper-forming segments in known amyloid folds

We next sought to develop metrics that describe the density of zipper segments in both the whole protein and specific protein domains. We termed Zif the fraction of protein segments that contain hexapeptides predicted to form zippers (see Methods). pAD, a domain specific metric, reports the total number of zipper segments per 75 residues in the sequence block with the most zipper segments. Hydrophobic residues pack well at the zipper interface and are also prominent in the membrane spanning regions of membrane proteins. Therefore, we implemented a masking procedure based on a segment’s GRAVY score to reduce interference between favorable zipper segments and transmembrane domains (see Methods). These metrics highlight proteins with a high number of zipper-prone segments, and we hypothesize that these proteins are prone to forming amyloid fibrils.

To investigate the behavior of these metrics, we examined the properties of protein sequences with experimentally-determined amyloid structures (S1 Table). This set of 36 unique protein sequences is populated mainly with human amyloids that have disease association but contains some examples across function and organism. The hexapeptide score distribution of these proven amyloid-forming proteins resembles that of the all-Hex set with no statistically significant differences (S7 Fig). The average Zif score is 0.15 ± 0.07 while the average pAD is 17.92 ± 8.70 (S9 Fig). Among the top ranked candidates are prominent amyloid proteins such as tau and α-synuclein. In the dataset of known amyloid proteins, functional amyloids (n = 7) have both Zif and pAD scores that are uniformly distributed across the dataset (n = 35), suggesting that both functional and pathogenic amyloids contain a proportionately equivalent number of zipper segments across their protein sequences and pAD regions. However, the way in which the adhesive interfaces come together to form the core is what dictates the behavior of that fibril species. The number of zipper segments is not alone indicative of how a protein will behave; this is further complicated by the fact that many of the identified proteins have fewer than 75 residues. When comparing Zifs within the fibril core with those of the overall polypeptide sequence, Zifs appeared to occur with higher frequency in the core, indicative of a potential correlation between the density of predicted zippers in a sequence and its formation of an amyloid fibril core. However, this trend proved to lack statistical significance (p = 0.0586) (S7b Fig). Thus far, however, there is no report suggesting the size of the amyloid core is related to pathogenicity [10].

We next looked at correlations between the protein’s properties and Zif score to gain a better understanding of features potentially correlated to zipper formation. We see strong correlations with Zif scores and a protein’s GRAVY and aliphatic index, both measures of hydrophobicity (S8a and S8c Fig). This makes sense given the strong positive correlations to hydrophobic and β-branched sequence composition and agrees with previously described literature on amyloid prone sequences.

We wanted to compare the performance of pAD on the set of observed amyloid structures. There are 13 observed polymorphs of the disease-associated, fully processed protein Islet Amyloid Polypeptide (IAPP). All observed cores overlap in sequence space (residues 34–70). pAD reports 17 of the 75 residues (0.23) are predicted to form zippers in the region 1–85 (multiple start positions with the same pAD score are reported) in unprocessed IAPP. If the window length is shortened to 40 residues, pAD reports a score of 14 out of 40 residues (0.35) are predicted to form zippers in the sequence region of residue 27–76. Both of these sets overlap with the observed regions of the fibril core.

In another example, we looked at Nup98, the large nucleoporin protein that is involved in transport across the nuclear pore complex but is found phase-separated in certain cancers and deposited in amyloid fibrils in Alzheimer’s disease where tau is also present [11]. Despite the large size of this protein, pAD reports the top domain between residues 34–128 with 27 of the 75 residues (0.36) predicted to have high propensity for forming zippers. The observed fibril core is between residues 84–124 and overlaps with the predicted pAD.

This dataset gives us an idea as to what these metrics look like for a set of proteins with known amyloid folds. However, given the small number of proteins in this set as well as their bias towards human disease state, we wondered what this type of analysis would look like at the proteome level.

### Evaluating zipper-forming propensity across the Saccharomyces cerevisiae proteome

We next wanted to understand the behavior of these metrics on a proteome level. As a starting point, we compared the behavior of the *Saccharomyces cerevisiae* proteome to the dataset of predicted yeast prion proteins in Alberti et al., 2009 [8]. The average Zif and pAD for the yeast proteome are 0.15 ± 0.05 and 19.5 ± 6.73, respectively. About 65% of the Alberti dataset lies above the proteome average for both metrics (S10 Fig).

**Fig 3:**
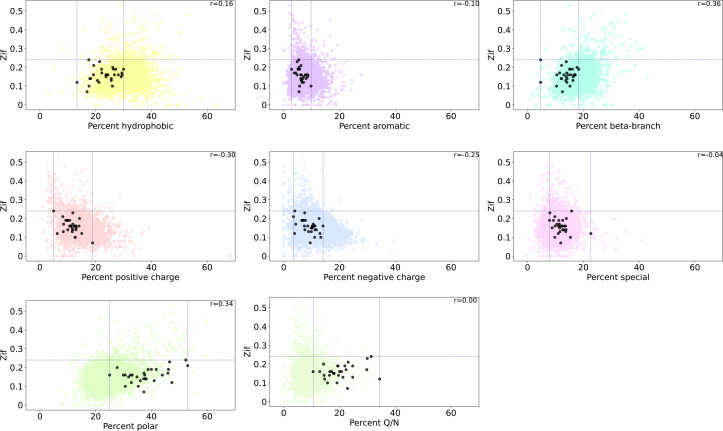
Correlation between Zif and percentage of amino acid type for proteins in the S. cerevisiae proteome. Black circles represent prospective yeast prion proteins presented in Aberti et al. [8] Pearson’s correlation coefficients are shown for each type or group of residues: (**a)** hydrophobic, (**b)** aromatic, (**c)** beta-branched, (d) positive charged, (e) negative charged, (**f)** special, (**g)** polar and (**h)** Q/N. Dashed lines represent minimum and maximum values for the subset of points indicated by black circles.

Zif is negatively correlated to high percentages of charged residue content in the protein sequence (Fig 3d-3e). This is expected as charged residues are not predicted to be compatible with adopting a steric zipper motif. Interestingly, there is a positive correlation with the percentage of polar residues in the sequence, but the positive correlation comes with increased percentage of S and T rather than Q and N (Figs 3g-3h and S11). This is unexpected given the fact that Q/N are typically enriched in known amyloid-based prions, a feature that was prominent amongst the Alberti data set. Thus, this approach likely highlights yeast amyloid proteins that are dissimilar to the Q/N-rich prions found in Alberti et al.

Gene Ontology (GO) term analysis on the top 100 candidate proteins ranked by Zif are enriched for proteins involved in flocculation, cell wall/organelle organization and protein metabolic processes. Similarly, top ranked candidates for pAD are enriched for proteins involved in cell adhesion in biofilm formation, flocculation, and cell wall organization. This is in agreement with amyloid screening studies that noted enrichment in cell wall proteins [12].

The top ranked protein according to pAD is the cell wall protein CRH1. This protein functions as a chitin transglycosylase and is upregulated upon an increase in temperature. pAD reveals a window between residue 299 and 374 with 67 predicted zipper segments (0.89) (Fig 1b). There are no experimental structures of this protein, but according to the AlphaFold3 (AF3) model, the N-terminal half of the protein is β-sheet rich with pLDDT scores > 90 indicating very high confidence in the prediction (Fig 1b) [13]. The C-terminal half, however, is unstructured and coincides with the putative amyloid domain (Fig 1b).

**Fig 4:**
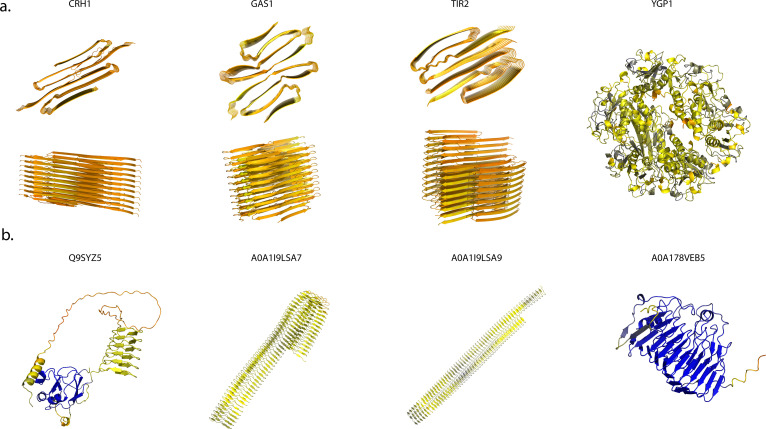
AlphaFold3 Structure Predictions. **a.**) AF3 models of predicted amyloid domains in top S. cerevisae pAD candidate proteins. **b.**) AF3 models of top pAD candidates in A. thaliana.

Wojciechowski et al. showed amyloid-like folds were predicted using AF3 under certain conditions [14]. To gain insight into the potential amyloid fold of CRH1, we modeled 20 copies of the putative amyloid domain. AF3 indeed produces a model with amyloid-like features; mainly ß-sheet rich, vertically stacked, with the correct distances present (Fig 4a). The 2-dimensional fold within the fibril core, however, is lost. It is these intricacies in the heterozipper arrangements that dictates how the fibril folds in on itself.

## Discussion

The identification of amyloid-forming segments across entire proteomes is a computationally intensive problem. However, we have capitalized on the advent of readily accessible ML protocols and a decade of scored zipper predictions generated by the ZipperDB webserver to implement an approach to this problem that relies on the ML-driven scoring of zipper-forming propensities for hexapeptides. This implementation is orders of magnitude faster than existing approaches for scoring zipper formation propensity, though it yields a simple predicted score rather than a structural model of a predicted zipper complex [9].

Given the speed of the updated ML-driven zipperDB, we considered it ideal to determine scores for all possible individual hexapeptide sequences. This analysis in turn yielded insights into potential biases in its scoring and enables proteome-wide characterization of zipper-forming propensities. As anticipated, an increasing number of hydrophobic residues led to a more stabilized zipper arrangement. These residues, especially Y, are known to stabilize amyloid fibrils due to favorable π-π stacking in the fibril core [6,15]. In our analysis, the average score for hexapeptide sequences remained relatively unchanged regardless of the number of aromatic residues present. It is possible that neighboring residues may not match the bulk created by large aromatic residues, which may create cavities in the core, destabilizing the arrangement. It should be noted that the scores produced by the presented method are based on classical energy-based optimizations. Newer energy functions as well as machine-learning based scoring methods can result in better predictions. In contrast, increasing numbers of branched polar residues in the hexapeptide sequence also produced more favorable zipper scores [6,15,16]. This was especially true for the β-branched amino acids (I, T, V), but also surprisingly true for serine. Conversely, we confirmed that an increased number of charged residues in the hexapeptide sequence results in a decrease in favorability. Unexpectedly, this decrease in favorability is present when adding charged residues to either the zipper interface or the solvent interface [3]. These discrepancies likely reflect the opportunity for improvement in the solvation parameters initially used to score the zipper structures, which were the basis for our ML training. A further limitation is our restriction of peptide backbones to a type-1 homozipper arrangement. Theoretically, however, our analysis could be expanded to all ten symmetry patterns of steric homozippers, as well as to heterozippers, where the mating sheets contain different amino acid sequences [6,16,17]. With our success in training of type-1 zippers, we believe that our structure-informed ML approach can be utilized to model and predict other steric zippers and the accompanied boost in computational speed could provide information on potential bias in the simulation protocol, especially for the implementation of the Rosetta score function.

Based on our analysis of zipper scores, we developed two predictive metrics that help assess proteome level zipper predictions. Zif describes the frequency of zipper segments on a whole protein, while pAD does so for a protein sub-domain. Our initial analysis performed on the *S. cerevisiae* proteome showed favorable agreement with previously identified putative prions or amyloids. The set of candidate yeast prions in the Alberti dataset are well distributed throughout the proteome according to both Zif and pAD, with about 70% residing above the proteome average for both metrics. However, while yeast prions are expected to have high Q and N content [7,8], high zipper propensity is not strictly correlated with Q/N content. This difference in propensity might reflect a residual bias in the energy function used for scoring in the original zipperDB [18].This is not entirely surprising, since that scoring function did not explicitly account for hydrogen bond networks that might stabilize zipper cores.

GO term analysis of the top 100 ranked proteins in yeast revealed enrichment for proteins that localize to the cell surface, extracellular space, and cell septum/bud scar. In particular, GO term analysis revealed groups of proteins involved in biofilm formation, flocculation, and cell wall remodeling. This is in agreement with literature from other fungal species where functional amyloids have been shown to be involved in processes at the cell wall such as hydrophobins in filamentous fungi [19–21]. Moreover, this is reminiscent of functional amyloids in bacterial species that are present at the cell wall such as the reproductive proteins of the chaplin family in *Streptomyces coelicolor* and the biofilm proteins present in *Escherichia coli* and Salmonella spp [22–26]. The proteins Gas1 and Ygp1 are both involved in cell wall function and have been shown to have amyloid properties in vitro and were ranked 24th and 72nd by pAD. Originally identified in a yeast screen using PSIA-LC-MALDI, these proteins form SDS-resistant puncta in yeast cells and were shown to have amyloid properties via the bacteria-based system C-DAG [12] (Fig 1b).

Intriguingly, among the top ranked protein domains is a family of serine-rich cell wall components. The TIR family (TIR1, TIR2, TIR3, TIR4) is upregulated upon an anaerobic shift in the environment where they move to the cell wall and replace existing cell wall proteins [27]. TIR2, TIR3, and TIR4 are all ranked within the top 10 with pAD scores of > 55 segments in the domain (0.73). The putative amyloid domain is predicted to start in the middle of the protein sequence, overlapping with a predicted unstructured region (AF3 pLDDT < 50).

In addition to our analysis of the yeast proteome, we pursued an additional model organism that is significantly understudied in amyloid biology, *Arabidopsis thaliana*. Proteome-wide zipper prediction in Arabidopsis revealed top ranked candidates are proteins that contain a high density of repeat segments and are predicted to have β-solenoid structures via AF3 (Fig 4b). This type of structure has been adopted by some prions and has amyloid characteristics. Most notably, the fungal prion Het-S uses a β-solenoid fold in its prion state to induce heterokaryon incompatibility [28,29]. Additionally, the bacterial amyloid, Curli, uses a β-solenoid fold as a structural component of a biofilm [30]. Most similar to the predicted fold, however, is the proposed amyloid-like β-solenoid fold of the ice nucleating proteins in bacteria [31–33]. This is supported by work from the Linquist lab, which showed potential prion behavior in proteins involved in the autonomous flowering pathway of this species [34]. Luminidependens (LD), Flowering Locus PA (FPA), and Flowering Locus CA (FCA) each possess prion-like properties. However, those proteins did not rank highly in our analysis likely due to the specific scoring of Q and N residues. Interestingly, those proteins were suggested to act as non-canonical prions due to lack of high molecular weight aggregates.

We have made our platform accessible to facilitate prediction and experimental validation of steric zipper forming segments and putative amyloidogenic proteins. We expect the speed and capacity of the new zipperDB to aid in the identification of proteins with a high proportion of zipper segments, which can narrow down the potential candidates for experimental testing. It remains to be seen whether a high density of zipper forming segments translates to high propensity to form amyloid fibrils *in vitro* and *in vivo*.

## Methods

### Zipper propensity prediction

We treated the prediction problem as a regression to minimize the differences between the score predicted for a given hexapeptide and the score calculated for it by the original ZipperDB protocol. The set of available training data, obtained from queries submitted to the ZipperDB database, contained a total of 4,144,232 hexamer sequences and their corresponding scores. Segments were randomly partitioned into the training, test, and validation set with 3,276,800 segments included in the training set, 733,184 segments in the validation set, and 134,248 in the test set (including 310 test amyloid segments will be 134,558).

We constructed a network whose structure was composed of three fully connected layers. In it, hexapeptide sequences were one-hot-encoded to a 6 × 20 array, and then passed onto a 120x120 fully connected hidden layer (S1a Fig). Each node was connected to every node in the output nodes through linear operations, so each node produced an output equivalent to:


$$y_{j}=\sum w_{i}x+b_{i}$$

After the first layer of the network, a rectified linear unit (ReLU) was adopted as the activation function to introduce non-linearity to the model as follows:


f(x)=max(0,x)

Outputs from the hidden layer were then passed through a 120 × 1 FC layer to produce a single number as a predicted score. Differences between predicted scores and those in the training set were minimized by reducing the mean squared loss (MSE) between the two. The loss function is calculated as follows:


$$\sum \frac{{\left(\text{P}-\text{T}\right)}^{\text{2}}}{\text{N}}$$

Where P is the predicted score, T is the original ZipperDB score, and N is the number of segments evaluated. Training converged after ~1,000 epochs, after which the loss score had decayed to ~0.2 REU [2](S1b Fig). With this model, we computed scores for all possible permutations of hexapeptide sequences, totaling 20^6^ = 64,000,000 segments. Scores for this number of segments were produced within 45 minutes on an NVIDIA GeForce RTX 3090 GPU. This included the time required to load the model, generate predictions, and write all outputs into an output CSV file.

We also evaluated the accuracy of the model by evaluating the performance on the test set. Among all 134,248 segments, 90.6% of segments fell within the error range of ±0.5 REU while 98.0% of segments fell in the range of ±1 REU, indicating a high degree of accuracy achieved by the model (S1d Fig). Additionally, we evaluated the network’s steric zipper prediction accuracy using a confusion matrix on the test set (S1c Fig). The original protocol contended that any hexamer that has a REU score ≤ -23 are predicted to form steric zippers. In this case, positive was considered as REU ≤ -23.0 while REU > -23.0 was considered negative. The confusion matrix was built as follows while the criteria is defined as:


$$Accuracy\;=\frac{TP+TN}{TP\;+\;TN\;+\;FP\;+\;FN}$$

$$Sensitivity\;=\frac{TP}{TP\;+\;FN}$$

$$Specificity\;=\frac{TN}{TN\;+\;FP}$$

Where TP is true positive, TN is true negative, FP is false positive, and FN is false negative. The model had an accuracy of 0.980, a sensitivity of 0.926, and a specificity of 0.992. It is worth noting that there was no clear distinction between some positive and negative segments. That is, for example, there was not a significant difference between a hexamer that had a score of -23.1 REU and another that has a score of -22.9 REU, though the latter was considered a negative while the former one a positive score.

To assess the impact of network architecture on zipper prediction capacity, we also evaluated a complex 1D convolution model in which we embedded the layer with an input dimension of 20 amino acids and an embedding layer of 800, after that we pass the parameters to a 1D convolution network with 5 layers with a final fully connected layer for output, resulting in a total of 1,282,401 parameters. The outcome of that effort indicated a similar accuracy on the test set as the simpler fully connected model we are focusing on in our manuscript (S13 Fig). We also furthered our exploration of the fully connected layer network by expanding the middle layer parameters to 14,400 instead of 120, or by adding another layer of dimension 120 as the 3rd layer. Those three networks showed a similar accuracy and evaluation criteria. In fact, expanding the hidden layer dimension from 60 to 500–2400 yielded only marginal performance improvement (S14 Fig). This suggested that the fully connected 3 layer network sufficed to reach the currently achieved plateau in prediction performance, with the heterogeneity and potential bias in the training data acting as a more fundamental limitation.

Overall, a simple FC network appears to replicate the time-consuming 3D profiling protocols of ZipperDB with a high accuracy and a significant increase in efficiency. Through the prediction of all possible permutations, we were then able to carry out proteome-level analysis of steric zipper formation within minutes or seconds.

### Calculation of zipper segment scores in Rosetta

To calculate scores for zipper segments whose sequence were not originally found in ZipperDB, we used same protocol described in Goldschmidt et al. [9] This approach was applied, in particular, to the calculation of scores for proline-containing segments, where a total of 1,563 proline containing sequences were generated randomly under the criteria of creating a near equal distribution of proline residues in all the six positions and the number of proline residues in the peptide was also balanced for statistical analysis. In PyRosetta, a 5 layer zipper peptide template structure was relaxed and energy minimized. Final Rosetta score was calculated by the total energies of one single peptide from the middle layer of the input template to avoid impacts from the environment.

### Calculated metrics

A number of metrics were calculated for each protein. Molecular weight was calculated as a sum of average isotopic masses of each amino acid in the sequence and the average isotopic mass of one H_2_O molecule (as in ExPASy ProtParam). GRand AVerage hydropathY (GRAVY) was calculated as a sum of Kyte-Doolittle hydrophobicity values across the sequence. Percent composition by amino acid and by type is the percent based off of the total sequence length. The amino acid types were split as follows: hydrophobic = (A, V, I, L, M), aromatic = (F, W, 0.5Y), positive = (H, K, R), negative = (D, E), polar = (N, Q, S, T, 0.5Y), special = (C, G, P). If an amino acid is in the sequence but not in the previous list, it was counted as non-canonical. Note tyrosine was assigned both polar and aromatic type and thus contributed a weight of 0.5 in each category.

Two additional metrics are calculated: the aliphatic index and instability index. The aliphatic index was defined as the relative volume of a protein occupied by aliphatic side chains (A, V, I, L) and was calculated as in Ikai, 1980 [35]. The instability index is an estimate of the relative stability of a protein in a test tube. A value < 40 was predicted to be stable and a value of > 40 predicted to be unstable [36].

All calculated error bars represent the standard deviation across data points, unless otherwise noted.

### Developed metrics

In addition to published metrics, we developed two protein level amyloid-specific metrics, Zif (Zipper fraction) and pAD (putative Amyloid-forming Domain) (S12 Fig). Zif represents the fraction of hexapeptides in the protein sequence that meet the threshold of qualifying as a zipper segment: any hexapeptide with a propensity score ≤ -23.

pAD corresponds to any stretch of 75 residues within a sequence that contains the highest density of zipper segments in a protein; this was calculated using a sliding window approach (S12 Fig). It reports the number of zipper segments present within the 75-residue window with the highest number of zipper segments. If a protein is smaller than 75 residues, the number of zipper segments in the whole protein was reported.

### Consideration of transmembrane helices

Initial analysis of top scoring Zif/pAD sequences revealed proteins that contained long, repetitive stretches of hydrophobic residues. Gene Ontology (GO) analysis revealed a large majority of these sequences encoded proteins with many transmembrane (TM) domains. Because of this, we implemented a masking protocol that allowed us to view proteome-level zipper predictions without the overrepresentation of transmembrane domain-containing proteins, alongside the raw zipper predictions. To identify putative transmembrane domains we used the protocol proposed by Kyte and Doolittle, 1982, where protein segments of 19 residues long with GRAVY scores greater than 1.6 are assumed to have a high chance of being transmembrane domains [37]. In the TM-masked set, any identified zipper segments that overlapped with an assumed transmembrane domain were not considered when calculating proteome-wide Zif and pAD metrics. We recognize that the TM-masked set may suffer from false negatives, but consider that integral membrane segments are likely to be sequestered by the membrane and thus may be less likely to freely form amyloid fibrils.

### Consideration of non-canonical amino acids, prolines, and splice variants

Sequences containing non-canonical amino acids were omitted from our training and calculations due to the sparse sampling of these with predictive modeling programs. Thus, any proteins that contain non-canonical amino acids were not explicitly scored for overall amyloid propensity, any calculated metrics (instability index, GRAVY, etc.), or developed metrics (Zif, pAD). Proteins containing non-canonical amino acids make up a small fraction of the total number of proteins in the proteome (0% in yeast, 0.02% in Arabidopsis). In addition, we noted that segments containing one or more proline residues tended to have high positive predicted score values. In fact, a survey of zipper propensity scores, calculated by Rosetta, from 1,563 proline-containing hexapeptide segments showed that any segment with one or more prolines was likely to have a high positive score (S15 Fig). Accordingly, we note a potential underlying bias associated with such segments and lowering the reliability of proline-associated predictions.

When analyzing whole proteomes, all splice variants were considered and plotted individually. For proteins in the amyloid atlas (S1 Table), the splice variant with the longest polypeptide sequence (i.e., unprocessed) was chosen, unless otherwise noted.

## Supporting information

S1 Fig Description and validation of the Structure-Informed Machine Learning Model (a) schematic diagram depicting architecture of the fully-connected neural network (b) The decay of loss during training (log scale) shows the model reached convergence after ~1000 epochs of training (c) Normalized confusion matrix shows the model achieved 98% accuracy. (d) Normalized error distribution performed on the test set shows that 98% of the error is within ± 1 REU of true score.
(TIF)

S2 Fig Relationship between amino acid composition and zipper propensity score. The number of (a) hydrophobic, (c) polar, and (f) β-branched residues are correlated with a more favorable zipper arrangement whereas the number of (d-e) charged and (g) special residues are correlated with a less favorable zipper score. Error bars represent the standard deviation.
(TIF)

S3 Fig Impact of individual amino acid counts on predicted amyloid propensity scores in hexapeptides. Averaged scores taken for hexapeptides with scores < 0. Error bars represent the standard deviation.
(TIF)

S4 Fig Impact of charged residues in a hexapeptide sequence on zipper propensity scores. As the number of charged residues or the magnitude of the total charge in the hexapeptide increases, the average propensity score increases. Error bars represent the standard deviation.
(TIF)

S5 Fig Charged residues at the zipper interface and the solvent interface have similar effects on the hexapeptide’s overall zipper propensity score. Error bars represent the standard deviation.
(TIF)

S6 Fig Mean zipper propensity scores for hexapeptides based on amino acid type and position. Schematic represents numeric positions based on Sawaya et al. 2007.[6] Error bars represent the standard deviation.
(TIF)

S7 Fig a) Score distribution for hexapeptides present in Amyloid Atlas. The distribution shape resembles that of all hexapeptides, with similar means. Black line represents the probability density function for the Amyloid Atlas dataset. The dataset mean is -21.23 ± 4.13 REU. b) zif comparison of the solved core vs full-length proteoform sequences for 9 notable amyloids. The cores, on average, have higher zif scores than their full length sequences but not statistically significant (p = 0.586).
(TIF)

S8 Fig Correlation between Zif and physical metrics for proteins with known amyloid structures (S1 Table). Pearsons’s correlation coefficients shown. Bounds represent minimum and maximum values.
(TIF)

S9 Fig Distribution of ranked Zif and pAD for proteins with solved amyloid fibril structures (see Amyloid Atlas). Red line denotes dataset mean.
(TIF)

S10 Fig Distribution of ranked Zif and pAD for proteins in S. cerevisiae proteome. Black dots represent prospective yeast prion proteins from Alberti dataset. Red line denotes dataset mean.
(TIF)

S11 Fig Correlation between Zif and amino acid content for proteins in the S. cerevisiae proteome. Pearsons’s correlation coefficients shown. Bounds represent minimum and maximum values. Black dots represent predicted yeast prions from Alberti dataset.
(TIF)

S12 Fig Metric Calculation. Zif is calculated by dividing the number of zipper segments by the total number of hexapeptide segments in the protein (magenta bar) PAD is calculated via a sliding window approach. The number of zipper segments is counted in 75 residue blocks. The reported number is the segment with the highest number of zipper segments. Cyan, blue, and purple bars represent the first three windows of alpha-synuclein (with 23, 24, 25 zipper segments, respectively). Green dots represent segments predicted to form zippers.
(TIF)

S13 Fig Comparison of network architectures for predicting zipper scores. Training and testing were performed on networks with three distinct structures, (a-c) a fully connected network with an expanded middle layer, (d-f) a fully connected network with an added layer, and (g-i) a convolutional network. (a, d, g) Decay of loss during training (log scale) is shown over a period of 2000 epochs. (b, e, h) Normalized confusion matrices show accuracy of each proposed network architecture. (c, f, i) Normalized error distributions show the typical network error in REUs.
(TIF)

S14 Fig Performance in amyloid propensity prediction by three fully connected neural network architectures with varying hidden layer sizes, all trained on the same set of ZipperDB data. The network architectures vary from a hidden layer size of 60 (a, b), 500 (c, d), and 2400 (e, f). Although training was carried out over 2000 epochs, all three models appear to reach convergence after ~50 epochs.
(TIF)

S15 Fig Average zipper propensity score as calculated by Rosetta from 1,563 proline-containing segments. (a) The impact of number of proline residues on the average zipper propensity score. (b) Mean zipper propensity scores for proline-containing segments based on position of proline in the hexapeptide sequence.
(TIF)

S1 Table Metrics for proteins in the Amyloid Atlas. (DOCX)
