# Supplementary figures and images for: Leveraging structure-informed machine learning for fast steric zipper propensity prediction across whole proteomes
Source: PLoS Comput Biol. 2025 Aug 25;21(8):e1013395. doi: 10.1371/journal.pcbi.1013395 (PMC12413084; doi:10.1371/journal.pcbi.1013395)

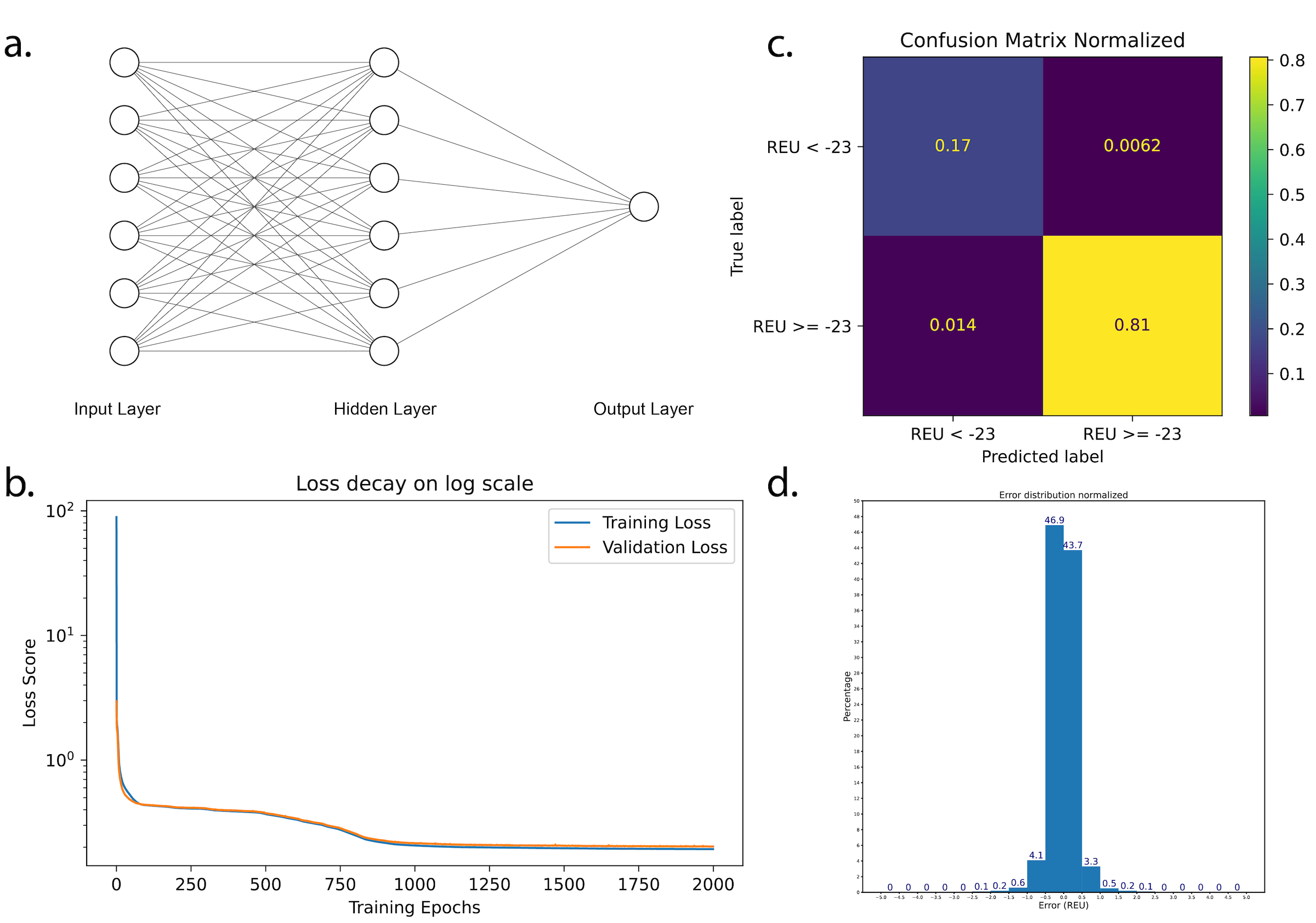

Supplement: S1 Fig — (d) Normalized error distribution performed on the test set shows that 98% of the error is within ± 1 REU of true score. (TIF) [file pcbi.1013395.s001.tif]

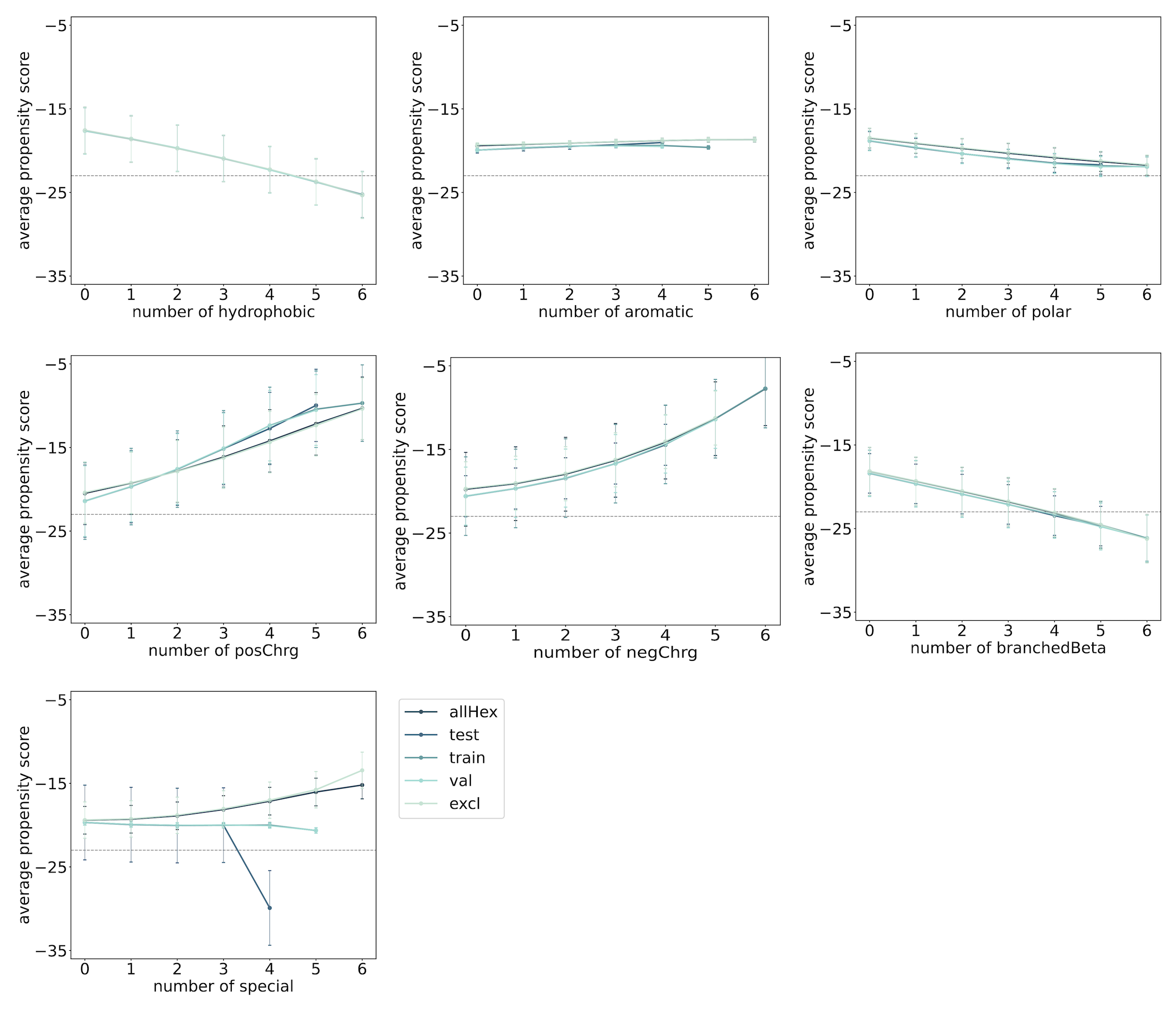

Supplement: S2 Fig — The number of (a) hydrophobic, (c) polar, and (f) β-branched residues are correlated with a more favorable zipper arrangement whereas the number of (d-e) charged and (g) special residues are correlated with a less favorable zipper score. Error bars represent the standard deviation. (TIF) [file pcbi.1013395.s002.tif]

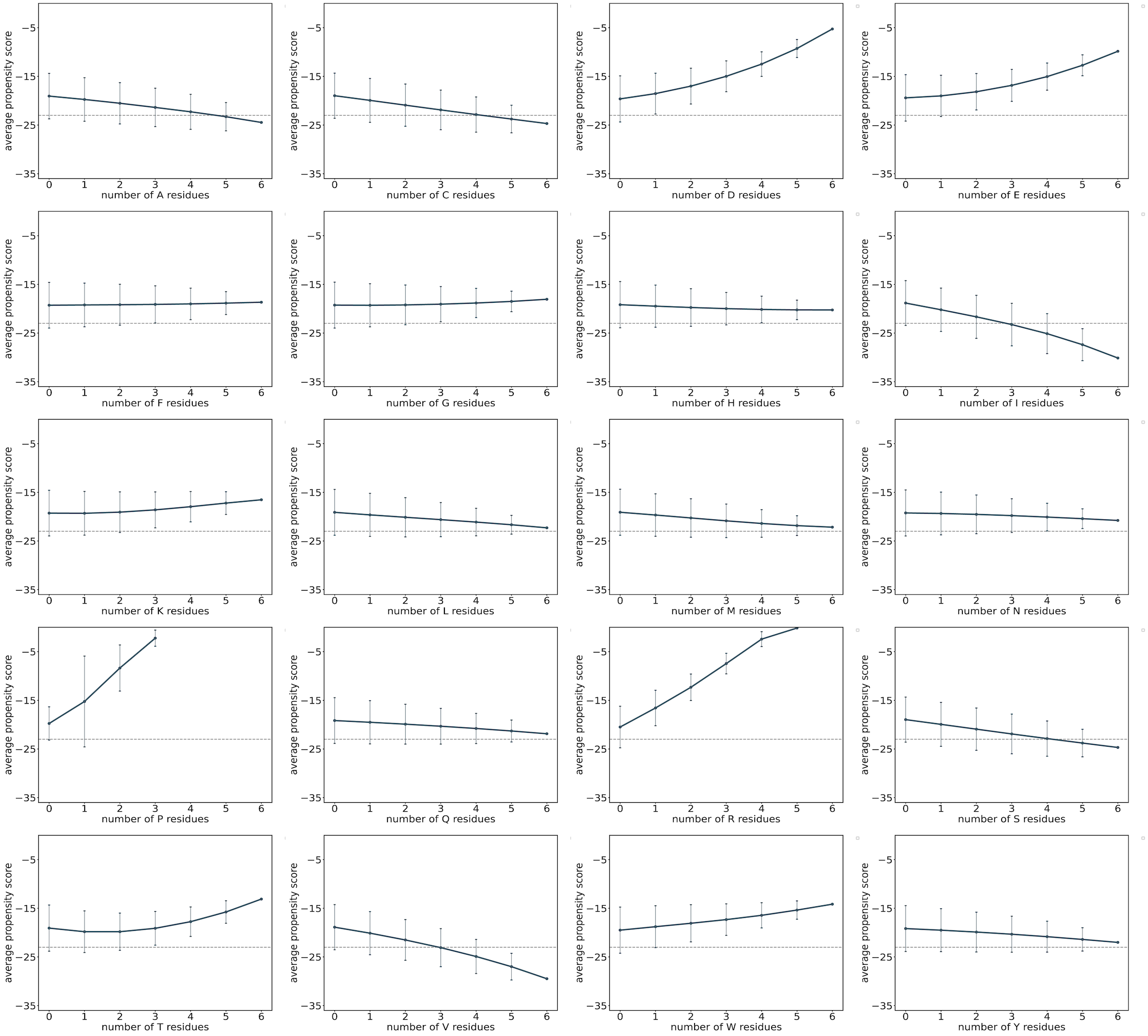

Supplement: S3 Fig — Averaged scores taken for hexapeptides with scores < 0. Error bars represent the standard deviation. (TIF) [file pcbi.1013395.s003.tif]

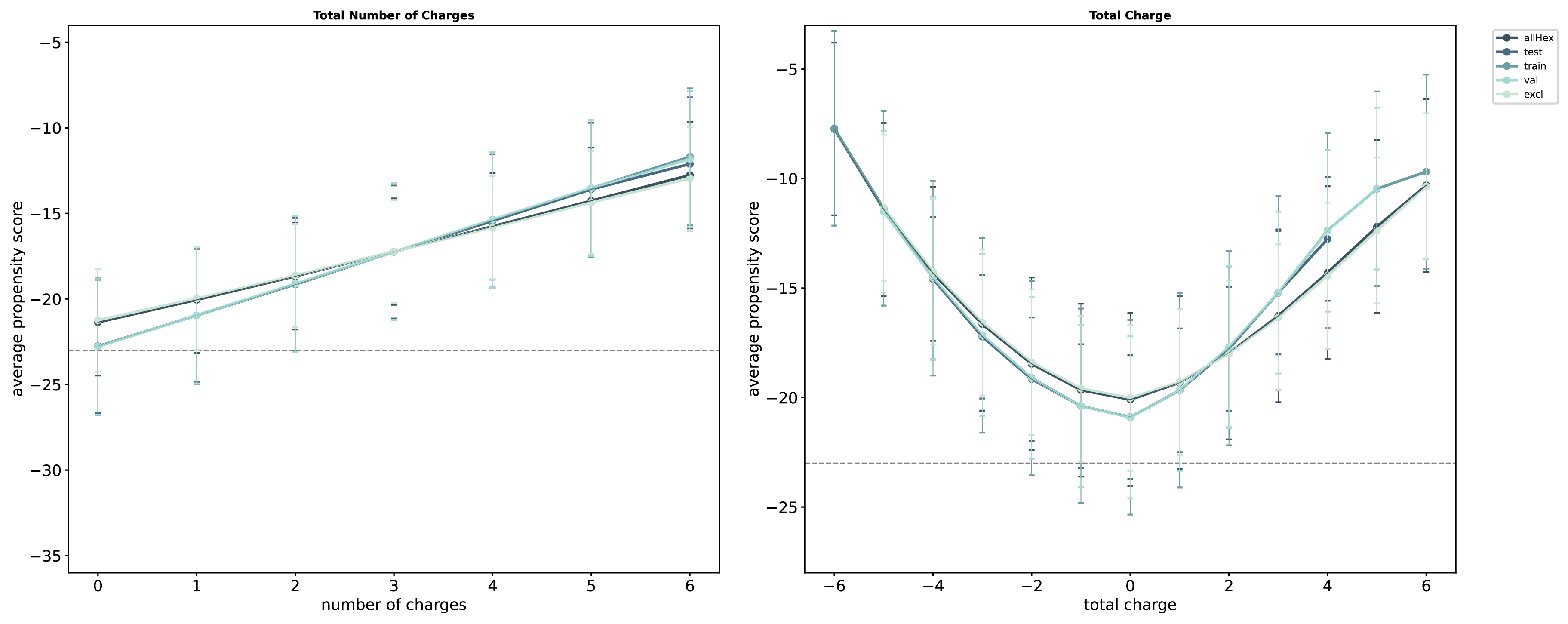

Supplement: S4 Fig — As the number of charged residues or the magnitude of the total charge in the hexapeptide increases, the average propensity score increases. Error bars represent the standard deviation. (TIF) [file pcbi.1013395.s004.tif]

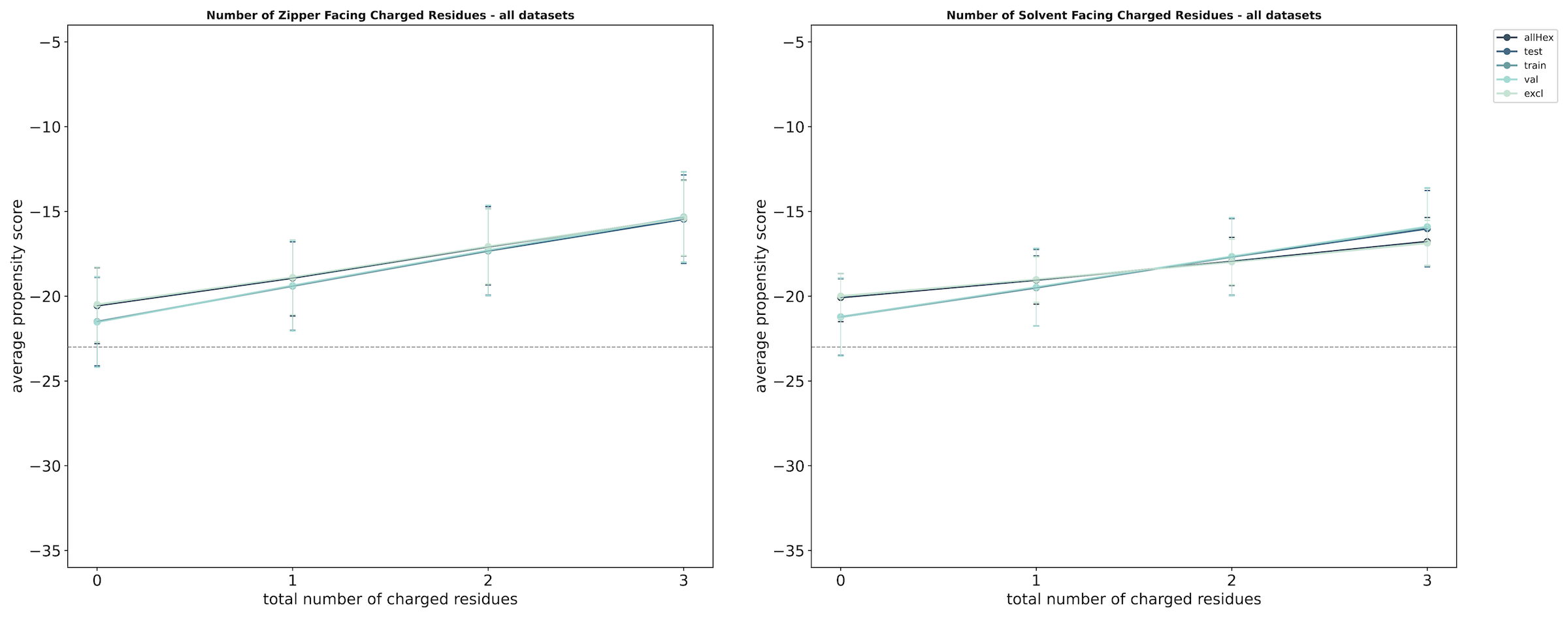

Supplement: S5 Fig — Error bars represent the standard deviation. (TIF) [file pcbi.1013395.s005.tif]

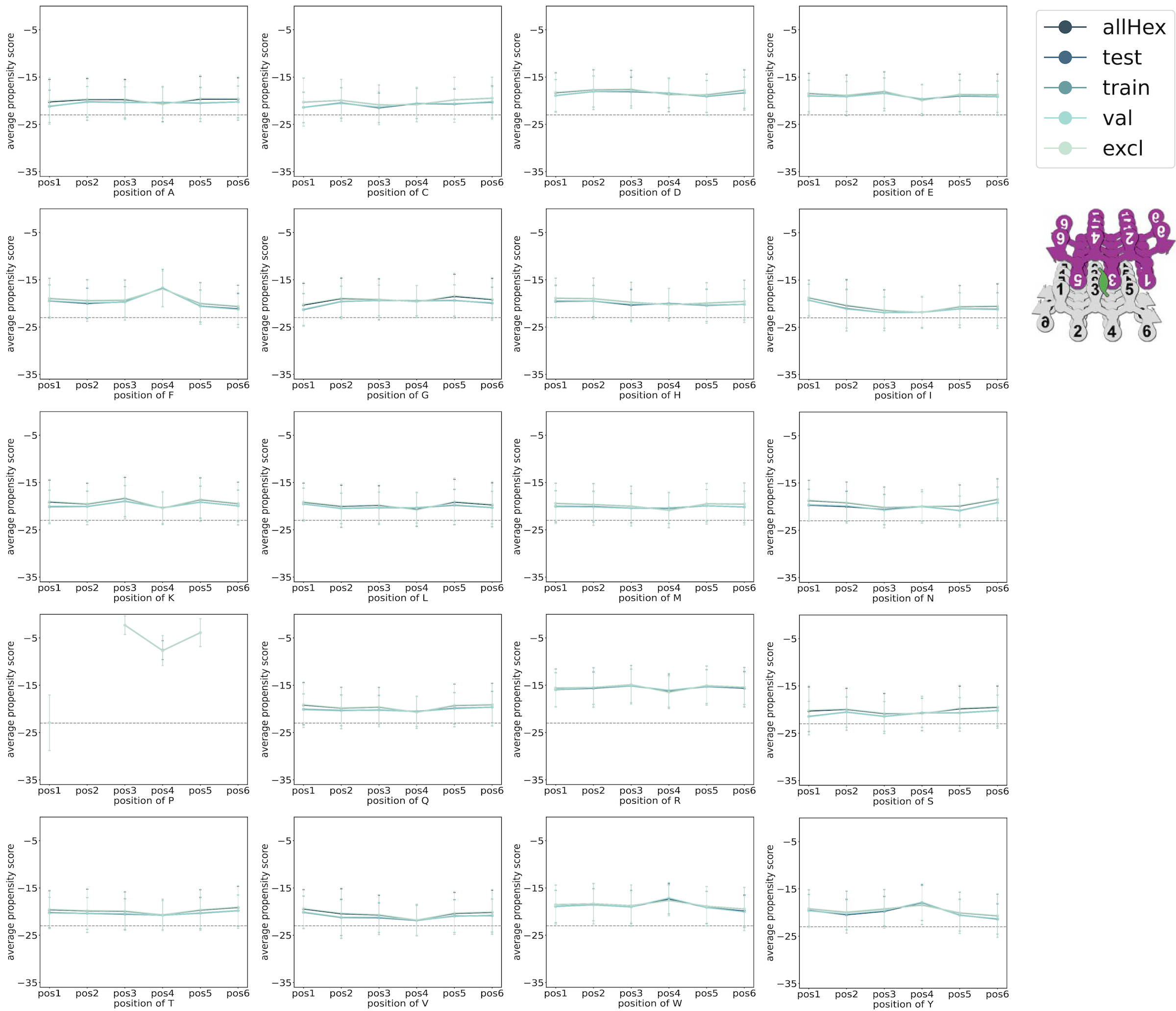

Supplement: S6 Fig — Schematic represents numeric positions based on Sawaya et al. 2007.[6] Error bars represent the standard deviation. (TIF) [file pcbi.1013395.s006.tif]

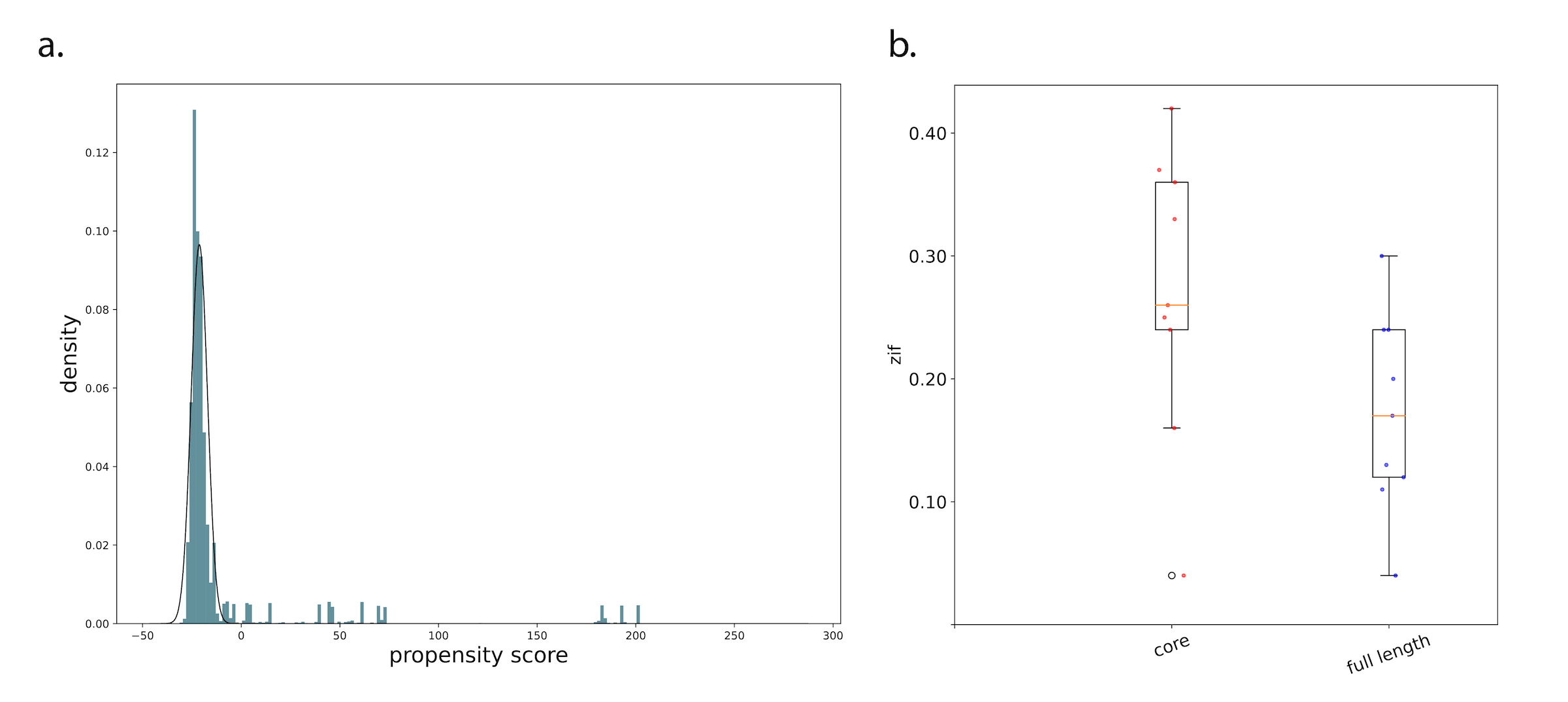

Supplement: S7 Fig — The dataset mean is -21.23 ± 4.13 REU. b) zif comparison of the solved core vs full-length proteoform sequences for 9 notable amyloids. The cores, on average, have higher zif scores than their full length sequences but not statistically significant (p = 0.586). (TIF) [file pcbi.1013395.s007.tif]

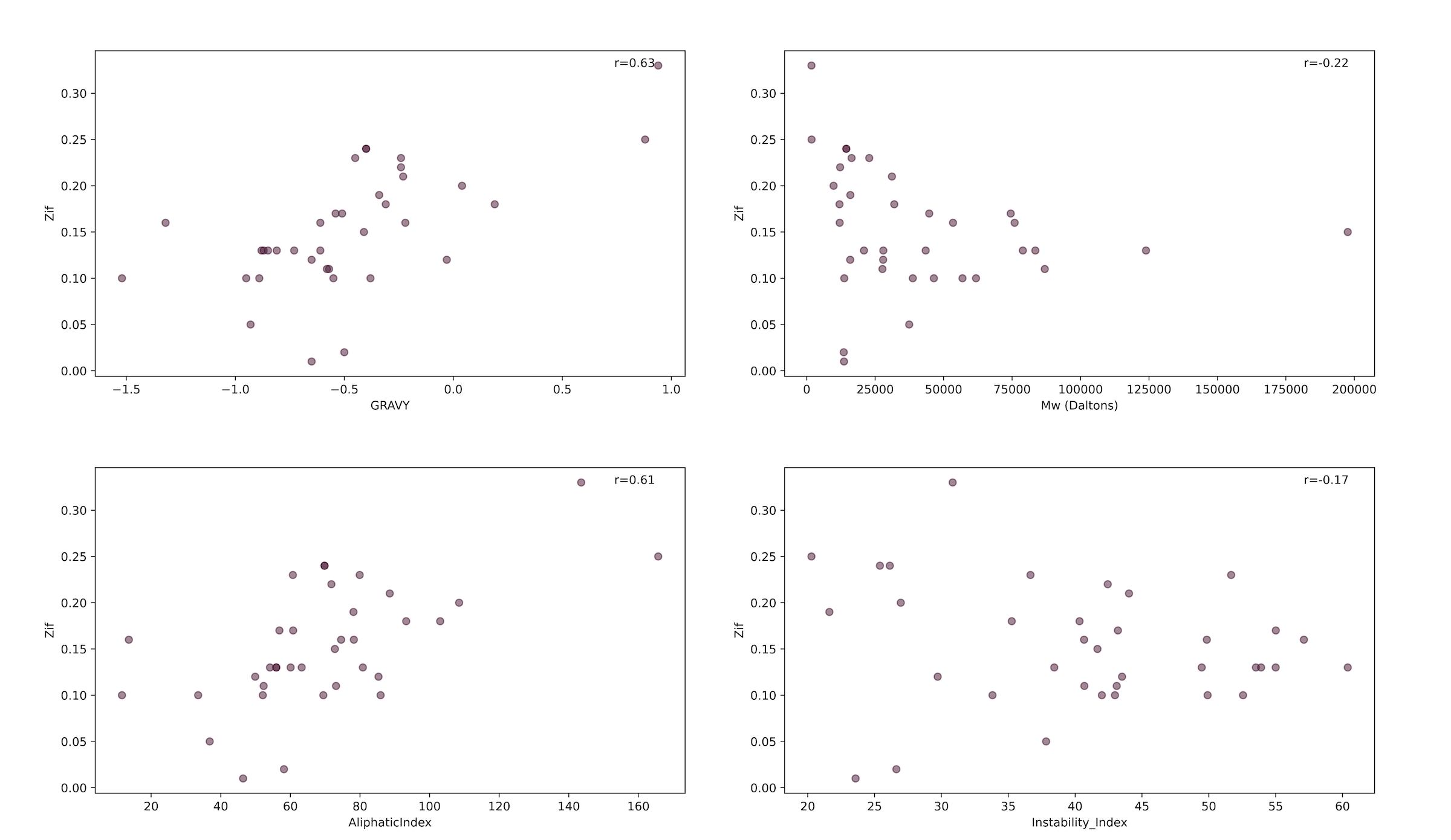

Supplement: S8 Fig — Bounds represent minimum and maximum values. (TIF) [file pcbi.1013395.s008.tif]

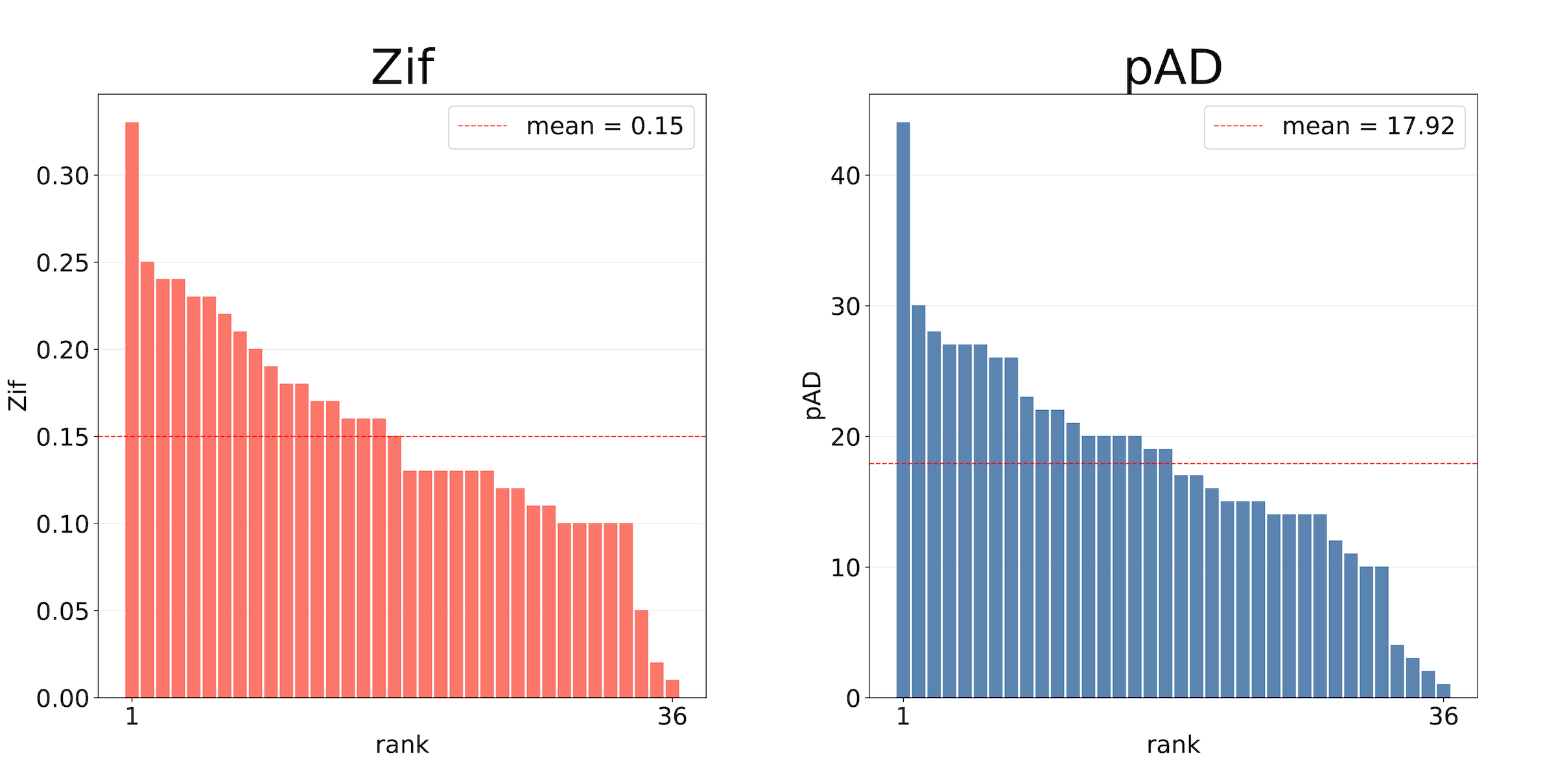

Supplement: S9 Fig — Red line denotes dataset mean. (TIF) [file pcbi.1013395.s009.tif]

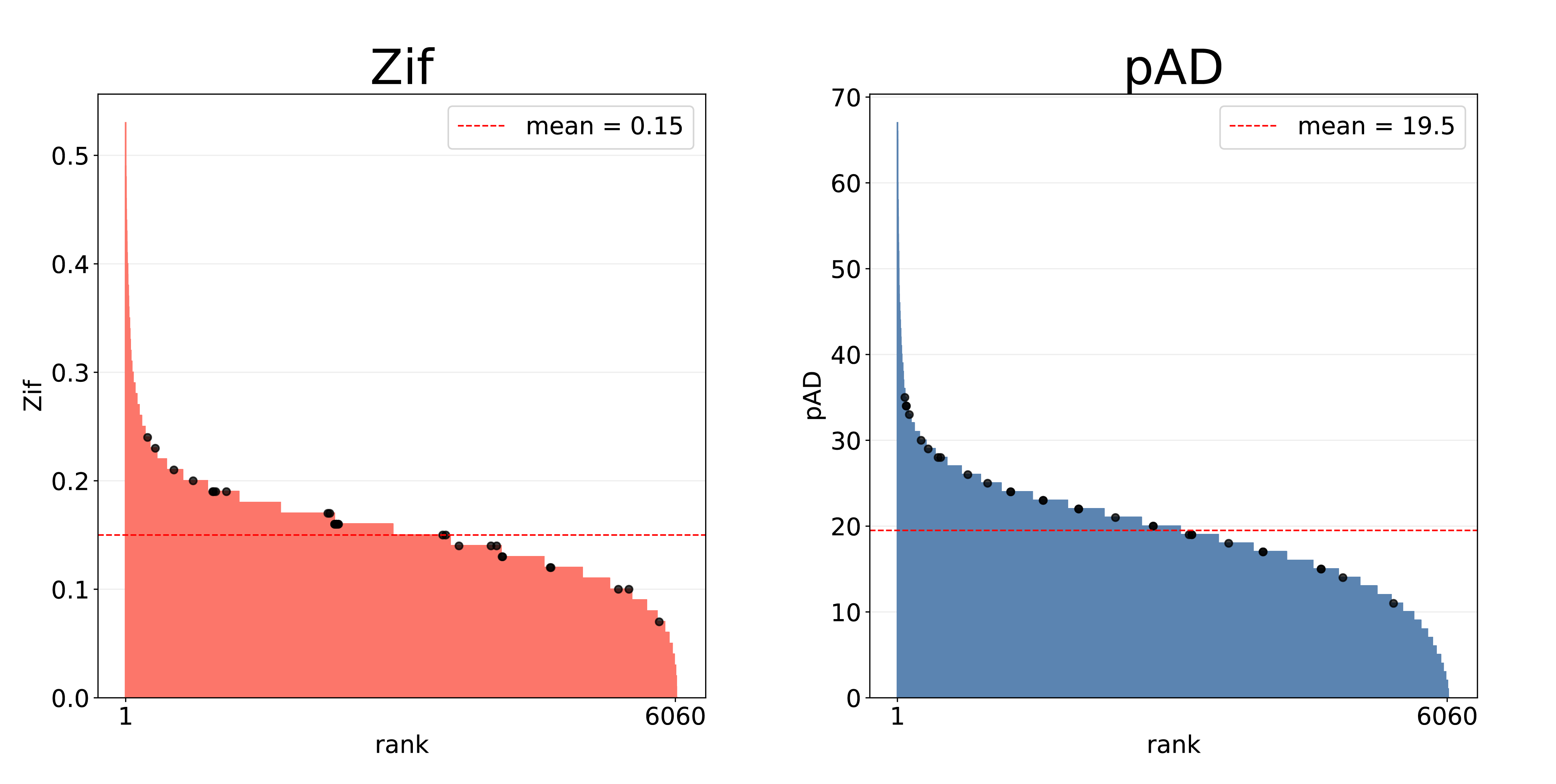

Supplement: S10 Fig — Black dots represent prospective yeast prion proteins from Alberti dataset. Red line denotes dataset mean. (TIF) [file pcbi.1013395.s010.tif]

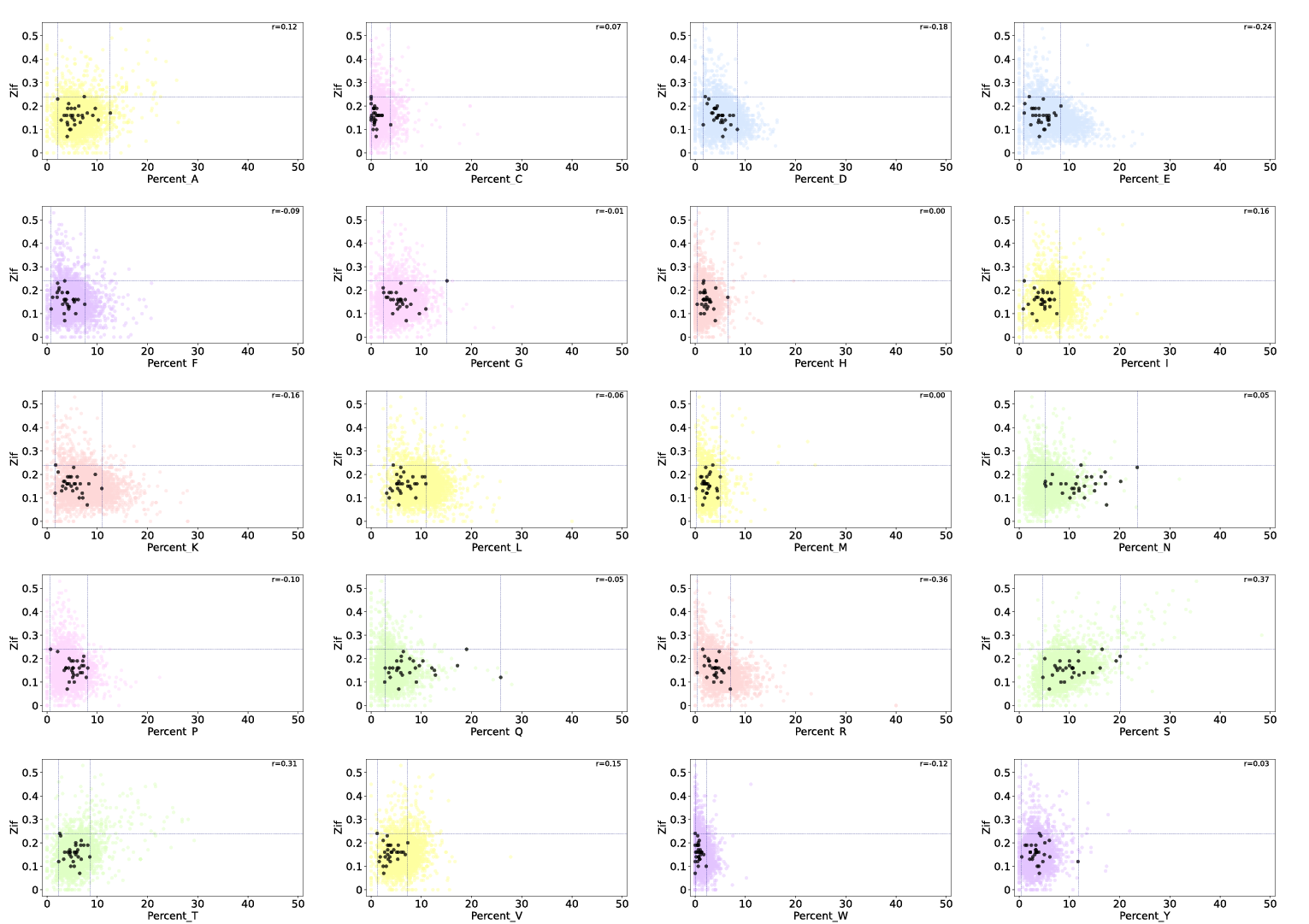

Supplement: S11 Fig — Bounds represent minimum and maximum values. Black dots represent predicted yeast prions from Alberti dataset. (TIF) [file pcbi.1013395.s011.tif]

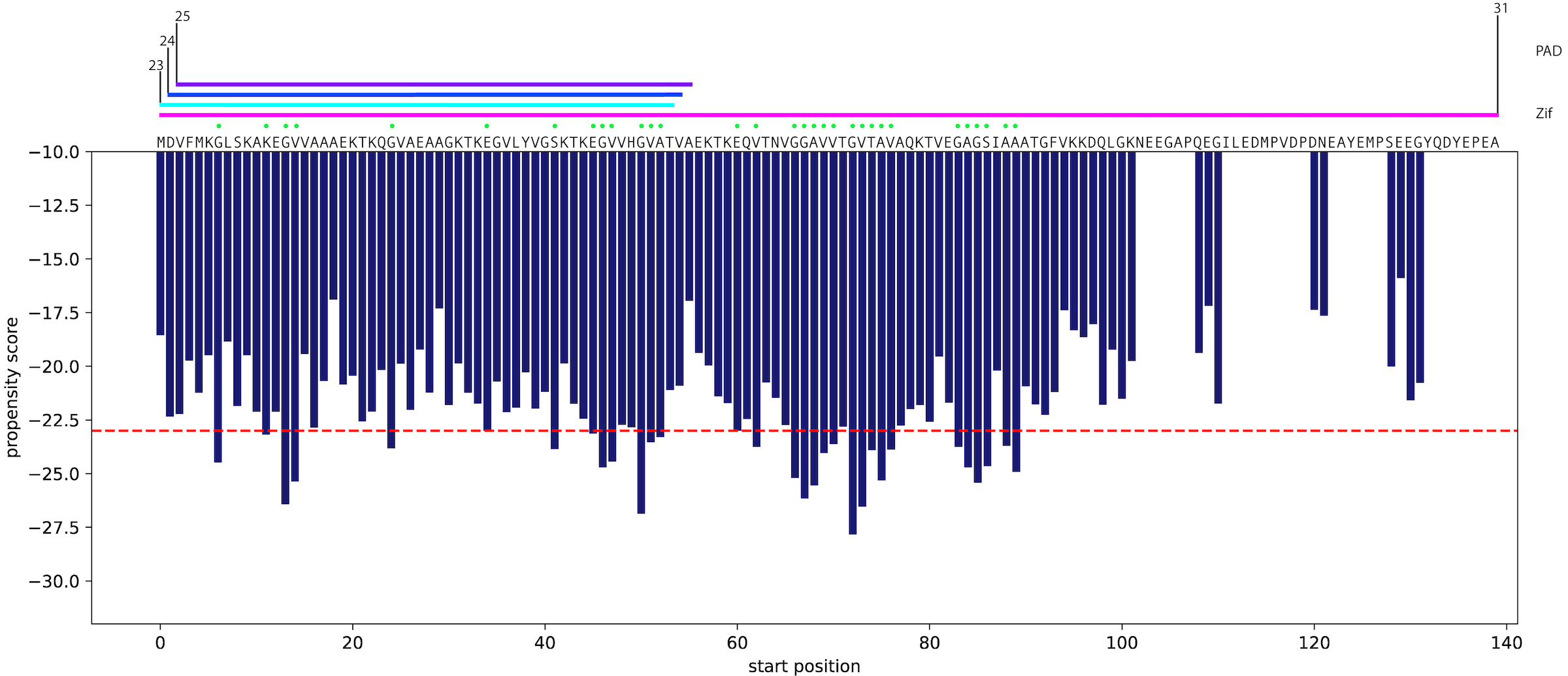

Supplement: S12 Fig — Zif is calculated by dividing the number of zipper segments by the total number of hexapeptide segments in the protein (magenta bar) PAD is calculated via a sliding window approach. The number of zipper segments is counted in 75 residue blocks. The reported number is the segment with the highest number of zipper segments. Cyan, blue, and purple bars represent the first three windows of alpha-synuclein (with 23, 24, 25 zipper segments, respectively). Green dots represent segments predicted to form zippers. (TIF) [file pcbi.1013395.s012.tif]

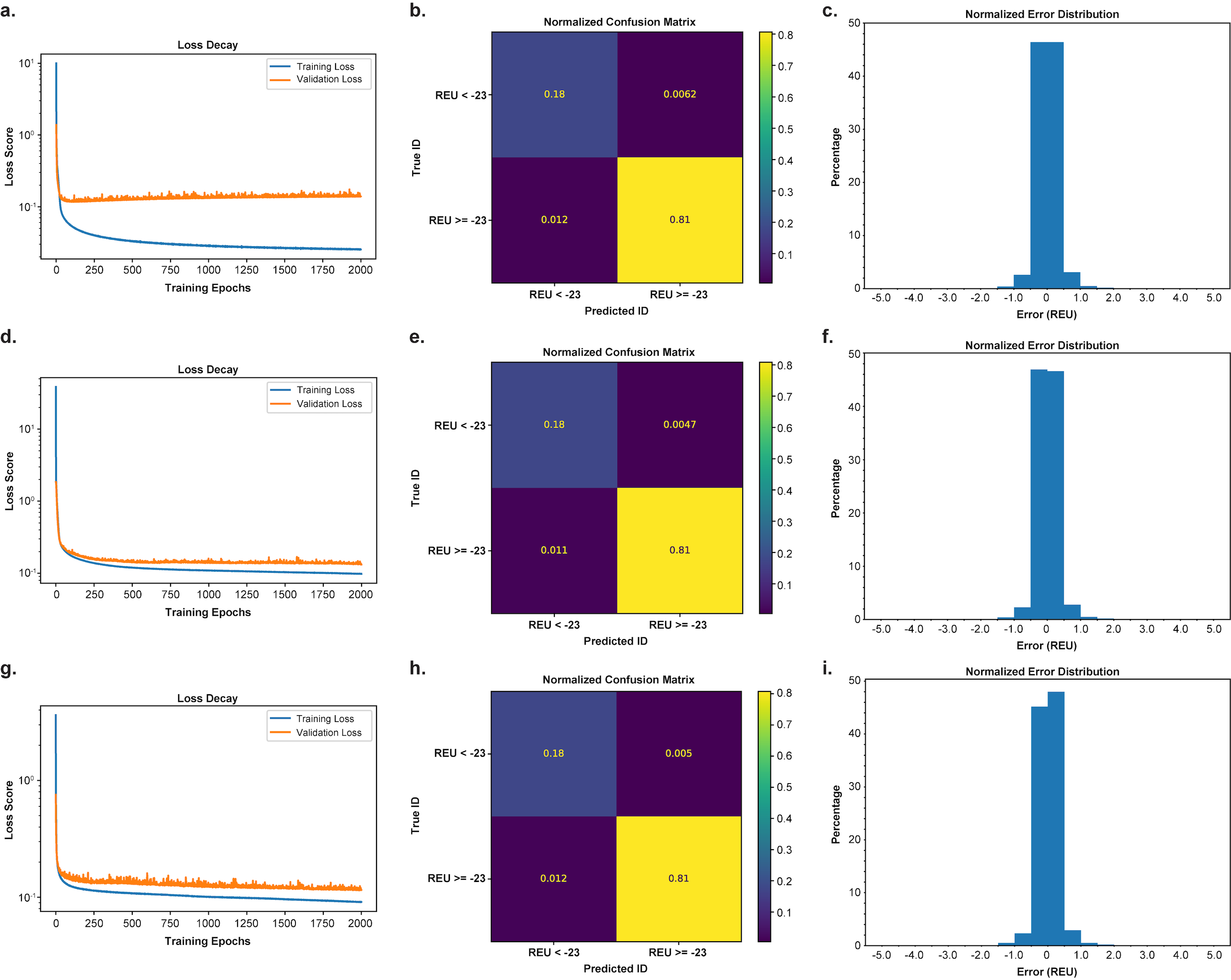

Supplement: S13 Fig — Training and testing were performed on networks with three distinct structures, (a-c) a fully connected network with an expanded middle layer, (d-f) a fully connected network with an added layer, and (g-i) a convolutional network. (a, d, g) Decay of loss during training (log scale) is shown over a period of 2000 epochs. (b, e, h) Normalized confusion matrices show accuracy of each proposed network architecture. (c, f, i) Normalized error distributions show the typical network error in REUs. (TIF) [file pcbi.1013395.s013.tif]

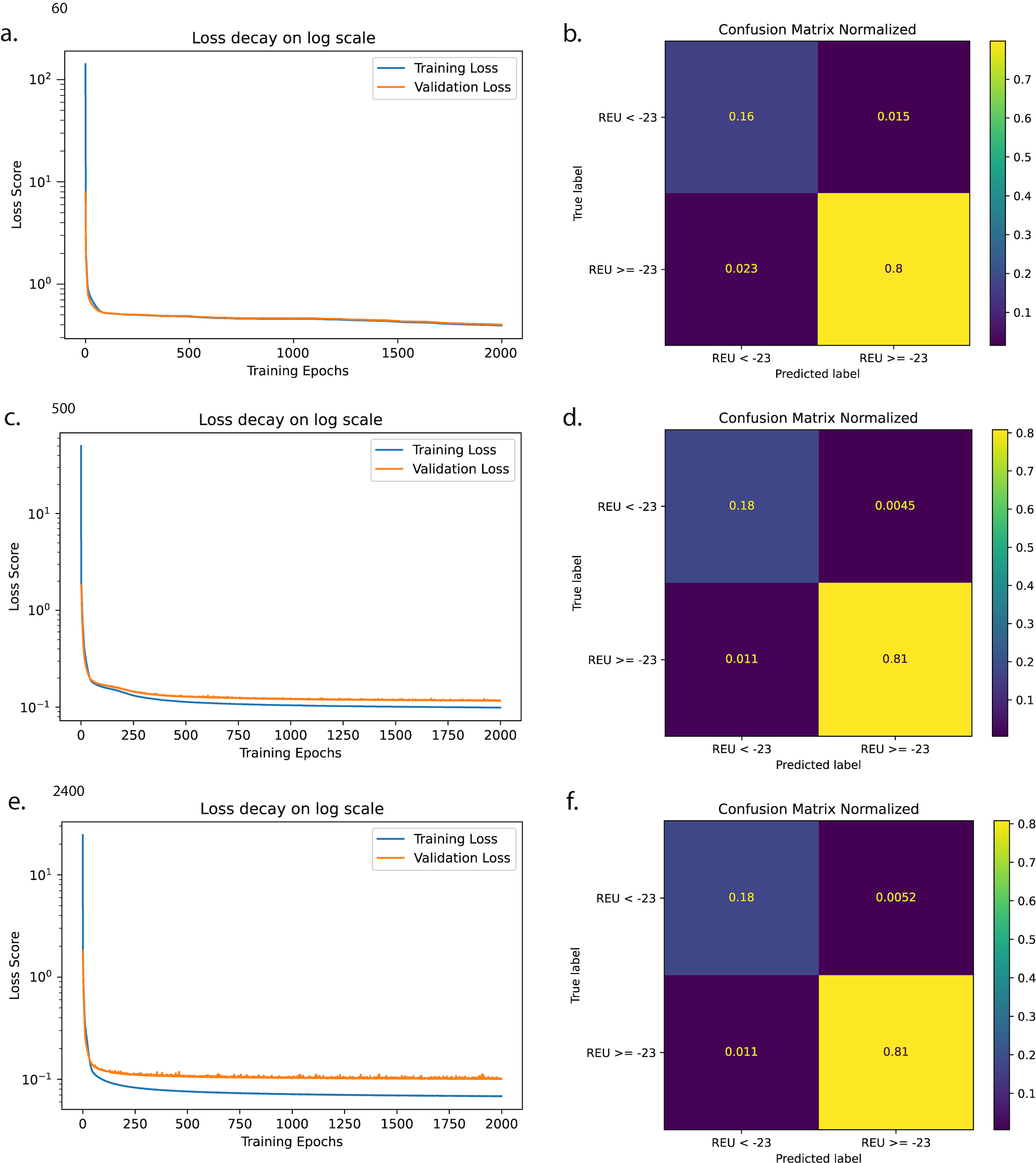

Supplement: S14 Fig — The network architectures vary from a hidden layer size of 60 (a, b), 500 (c, d), and 2400 (e, f). Although training was carried out over 2000 epochs, all three models appear to reach convergence after ~50 epochs. (TIF) [file pcbi.1013395.s014.tif]

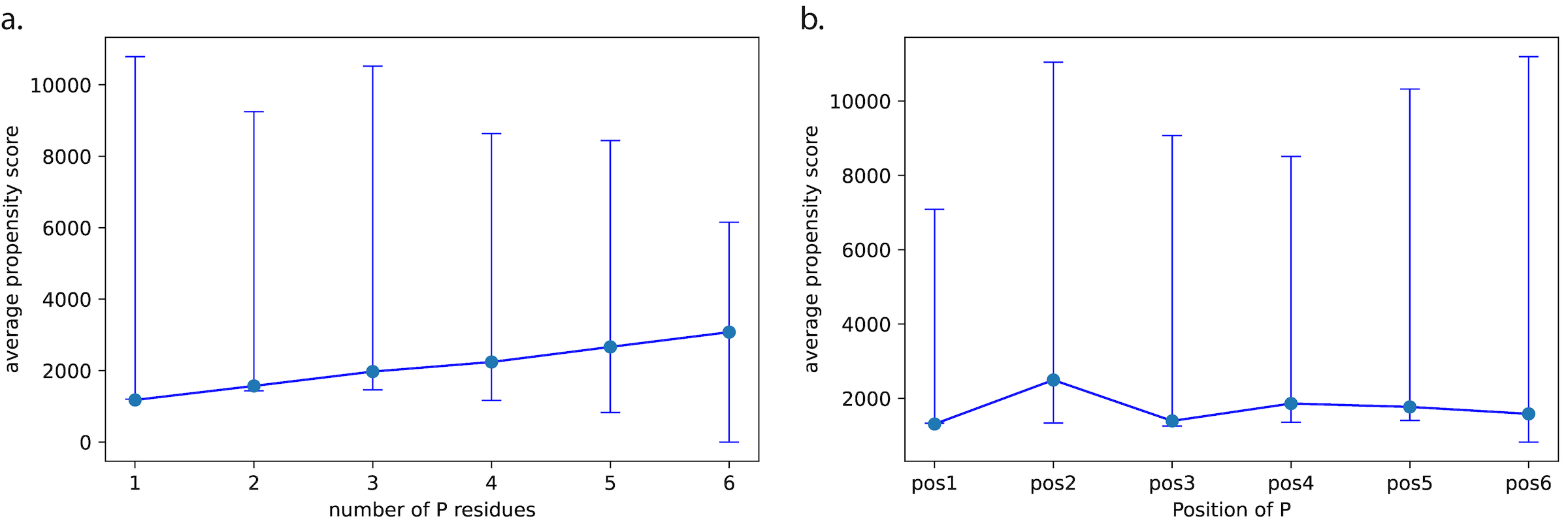

Supplement: S15 Fig — (a) The impact of number of proline residues on the average zipper propensity score. (b) Mean zipper propensity scores for proline-containing segments based on position of proline in the hexapeptide sequence. (TIF) [file pcbi.1013395.s015.tif]
