# Supplementary material for: Leveraging structure-informed machine learning for fast steric zipper propensity prediction across whole proteomes
Source: PLoS Comput Biol. 2025 Aug 25;21(8):e1013395. doi: 10.1371/journal.pcbi.1013395 (PMC12413084; doi:10.1371/journal.pcbi.1013395)
Supplement: S1 Table — (DOCX) [file pcbi.1013395.s016.docx]

**S1 Table:** Metrics for proteins in the Amyloid Atlas

| **Accession ID** | **Gene** | **Predicted TMD** | **Zif** | **pAD** |
| --- | --- | --- | --- | --- |
| P69020 | AUR31 | 0 | 0.33 | 4 |
| P82042 | UPE35 | 0 | 0.25 | 3 |
| P37840 | SNCA | 0 | 0.24 | 27 |
| O14960 | LECT2 | 0 | 0.23 | 22 |
| P0DOX8 | IGGL | 0 | 0.23 | 21 |
| P01699 | IGLV1_44 | 0 | 0.22 | 19 |
| Q9NUM4 | TMEM106B | 1 | 0.21 | 30 |
| P10997 | IAPP | 1 | 0.2 | 17 |
| P00441 | SOD1 | 0 | 0.19 | 14 |
| Q03689 | het_s | 0 | 0.18 | 20 |
| P01308 | INS | 0 | 0.18 | 14 |
| Q9VSR3 | orb2 | 0 | 0.17 | 26 |
| Q13148 | TARDBP | 0 | 0.17 | 20 |
| P35637 | FUS | 0 | 0.16 | 26 |
| Q13546 | RIPK1 | 0 | 0.16 | 20 |
| P01714 | IGLV3_19 | 1 | 0.16 | 17 |
| P52948 | NUP98 | 0 | 0.15 | 27 |
| O14776 | TCERG1 | 0 | 0.13 | 44 |
| P10636 | MAPT | 0 | 0.13 | 28 |
| P23727 | PIK3R1 | 0 | 0.13 | 22 |
| Q92734 | TFG | 0 | 0.13 | 19 |
| P04925 | Prnp | 1 | 0.13 | 16 |
| P01275 | GCG | 0 | 0.13 | 12 |
| P04273 | PRNP | 1 | 0.12 | 15 |
| P02766 | TTR | 1 | 0.12 | 11 |
| P05067 | APP | 1 | 0.11 | 20 |
| P04156 | PRNP | 2 | 0.11 | 15 |
| Q92804 | TAF15 | 0 | 0.1 | 23 |
| O14979 | HNRNPDL | 0 | 0.1 | 15 |
| P09651 | HNRNPA1 | 0 | 0.1 | 14 |
| Q9Y572 | RIPK3 | 0 | 0.1 | 14 |
| P61769 | B2M | 1 | 0.1 | 10 |
| P22626 | HNRNPA2B1 | 0 | 0.05 | 10 |
| P0DJI8 | SAA1 | 2 | 0.02 | 2 |
| P05367 | Saa2 | 3 | 0.01 | 1 |
